# Observing another in pain facilitates vicarious experiences and modulates somatosensory experiences

**DOI:** 10.3389/fnhum.2014.00631

**Published:** 2014-08-20

**Authors:** Sophie Vandenbroucke, Geert Crombez, Tom Loeys, Liesbet Goubert

**Affiliations:** ^1^Department of Experimental-Clinical and Health Psychology, Ghent UniversityGhent, Belgium; ^2^Department of Data Analysis, Ghent UniversityGhent, Belgium

**Keywords:** vicarious pain responders, synesthesia, visuotactile, multimodal, somatic contagion, pain contagion

## Abstract

**Objective:** This study investigated whether individuals reporting vicarious pain in daily life (e.g., the self-reported vicarious pain group) display vicarious experiences during an experimental paradigm, and also show an improved detection of somatosensory stimuli while observing another in pain. Furthermore, this study investigated the stability of these phenomena. Finally, this study explored the putative modulating role of dispositional empathy and hypervigilance for pain.

**Methods:** Vicarious pain responders (i.e., reporting vicarious pain in daily life; *N* = 16) and controls (*N* = 19) were selected from a large sample, and viewed videos depicting pain-related (hands being pricked) and non-pain related scenes, whilst occasionally experiencing vibrotactile stimuli themselves on the left, right or both hands. Participants reported the location at which they felt a somatosensory stimulus. We calculated the number of vicarious errors (i.e., the number of trials in which an illusionary sensation was reported while observing pain-related scenes) and detection accuracy. Thirty-three participants (94.29%) took part in the same experiment 5 months later to investigate the temporal stability of the outcomes.

**Results:** The vicarious pain group reported more vicarious errors compared with controls and this effect proved to be stable over time. Detection was facilitated while observing pain-related scenes compared with non-pain related scenes. Observers' characteristics, i.e., dispositional empathy and hypervigilance for pain, did not modulate the effects.

**Conclusion:** Observing pain facilitates the detection of tactile stimuli, both in vicarious pain responders and controls. Interestingly, vicarious pain responders reported more vicarious errors during the experimental paradigm compared to controls and this effect remained stable over time.

## Introduction

Evidence reveals that similar brain areas are activated when observing pain in another and when experiencing pain ourselves (Jackson et al., 2006; Bufalari et al., 2007; Gu and Han, 2007; Keysers et al., 2010; Lamm and Singer, 2010; Corradi-Dell'Acqua et al., 2011; Lamm et al., 2011). These observations are intriguing as they show that actual nociceptive input is not necessary to activate those brain regions which are also activated when being in pain.

While most individuals feel empathic and distressed in response to the observation of another in pain (Goubert et al., 2005), a minority actually reports vicarious somatosensory experiences. Percentages range from 1.6% for vicarious touch (Banissy et al., 2009), 16.20% for vicarious pain in amputees (Fitzgibbon et al., 2010a), to 6.61% (Vandenbroucke et al., 2013, study 1), 22.90% (Vandenbroucke et al., 2013, study 2) and 30% for vicarious pain in a general population (Osborn and Derbyshire, 2010). The variability is probably dependent upon the criteria used for categorizing individuals as vicarious pain responders.

Little research is available regarding the robustness of vicarious experiences and whether these change within individuals over time. Recruitment of participants reporting vicarious experiences and pain is largely based upon self-reports, mainly using questionnaires or interviews (Banissy and Ward, 2007; Fitzgibbon et al., 2010a; Vandenbroucke et al., 2013). For example, individuals are asked to rate whether they experience vicarious sensations in specific situations or in daily life. Based upon these ratings, participants are selected in a second phase, to take part for example in neuroimaging (e.g., Osborn and Derbyshire, 2010) or in an experimental study (e.g., Vandenbroucke et al., 2013). An implicit assumption of this recruitment procedure is that vicarious experiences are stable across time and across situations. However, to our knowledge, no study has examined whether the report of vicarious experiences is stable over time.

Also, little is known about the conditions affecting this phenomenon (but see Fitzgibbon et al., 2010b, 2012; Vandenbroucke et al., 2014). Many moderators have been proposed, but research is needed to corroborate these ideas and to replicate preliminary findings. For example, Vandenbroucke et al. (2013) showed that vicarious pain responders reported more vicarious pain experiences compared with controls. Hypervigilance to pain, or the over-alertness to pain-related information (as measured by a self-report instrument) moderated this effect, with vicarious pain responders reporting less vicarious errors when more hypervigilant for pain. However, in general, only few vicarious pain experiences occurred in this study (Experiment 1: 2.7%; Experiment 2: 0.88%) suggesting that it is a rare phenomenon, occurring in only some participants. Interestingly, in the study of Osborn and Derbyshire (2010), the most frequent descriptor that was selected from the McGill Pain Questionnaire to describe vicarious pain was “tingling.” Therefore, it is unclear whether vicarious pain responders in the general population (e.g., undergraduates) do experience vicarious pain or rather vicarious vague sensations while observing another in pain. Furthermore, observing somatosensation in another may not only induce vicarious somatosensory experiences, but may also influence the detection of tactile stimuli (Cardini et al., 2013; Gillmeister, 2014). For example, observing a face being touched enhances tactile perception on the face (Serino et al., 2008). In this context, the modulation of somatosensory experiences may represent a less extreme variant of the elicitation of “illusory” experiences when observing another in pain. Common pathways exist in experiencing touch and pain, such as multimodal neurons which both respond to nociceptive and tactile inputs (Le Bars, 2002). An overlap between processing nociceptive and non-nociceptive events has also been observed by Mouraux et al. (2011). These authors stress that the brain responses typically triggered by nociceptive stimuli are largely the result of both multimodal neural- and somatosensory-specific activities, rather than the result of nociceptive-specific neural activities. Of particular interest to this study, Vandenbroucke et al. (2014) showed that the observation of pain in others resulted in vicarious tactile experiences, which further attests to the interplay in processing touch and pain. The aims of this study were three-fold. First, we investigated whether the experience of vicarious somatosensory experiences and the detection of subtle somatosensory stimuli while observing another in pain differs in a group of vicarious pain responders vs. controls. Second, we examined whether these outcomes remain stable over time. Finally, the modulating role of dispositional empathy and hypervigilance for pain was explored. Using a variant of the crossmodal congruency task (see Vandenbroucke et al., 2013), vicarious pain responders (i.e., those who report vicarious pain during daily life; *N* = 16) and controls (i.e., those not reporting vicarious pain during daily life; *N* = 19) were presented videos of two categories, i.e., videos of pain-related situations (hands being pricked) and videos of non-pain related situations (e.g., sponge being pricked, hand approached by another hand). Participants occasionally received non-painful subtle vibrotactile stimuli themselves on the left, right or both hands. In 25% of the trials no vibrotactile stimulus was presented. Participants were instructed to report as rapidly as possible the spatial location of the administered somatosensory stimuli. Five months later, the same participants were invited again to execute the experiment a second time. First, we hypothesized that vicarious pain responders would report more bodily illusions in response to the observation of pain (vicarious experiences) than controls. As such we wanted to replicate the findings of Vandenbroucke et al. (2013). However, an important difference with this study is the inclusion of tingling instead of painful somatosensory stimuli. The use of vibrotactile instead of electrocutaneous “pricking” stimuli (see Vandenbroucke et al., 2013) may lead to an increase in vicarious experiences, as vicarious sensations has been most often described by vicarious pain responders as “tingling” rather than painful (Osborn and Derbyshire, 2010). Second, we examined the stability of vicarious experiences: if the experience of vicarious sensations is a robust and reliable phenomenon, comparable results should be obtained regarding the number of vicarious experiences at both time moments. Third, we expected that the observation of pain-related visual scenes would modulate the detection of vibrotactile stimuli compared with non-pain related scenes. In particular, we expected a crossmodal congruency effect (CCE) in which more tactile acuity is observed when the visual and tactile stimuli are spatially congruent. We hypothesized this CCE effect to be dependent upon the type of visual information (pain-related vs. non-pain related). As pain-related visual stimuli may facilitate detection of somatosensory stimuli, a higher CCE was expected when pain-related visual stimuli were shown as compared to non-pain related visual stimuli. We expected this CCE during pain-related videos to be most pronounced in the vicarious pain responder group. Relatedly, we explored whether the observation of pain-related scenes would result in neglect errors (i.e., only reporting the site congruent to the visual information when both hands are stimulated). Fourth, we explored whether dispositional empathy and hypervigilance to pain moderates the effects upon vicarious experiences and the detection of tactile stimuli.

## Materials and methods

### Participants

**Figure 1:**
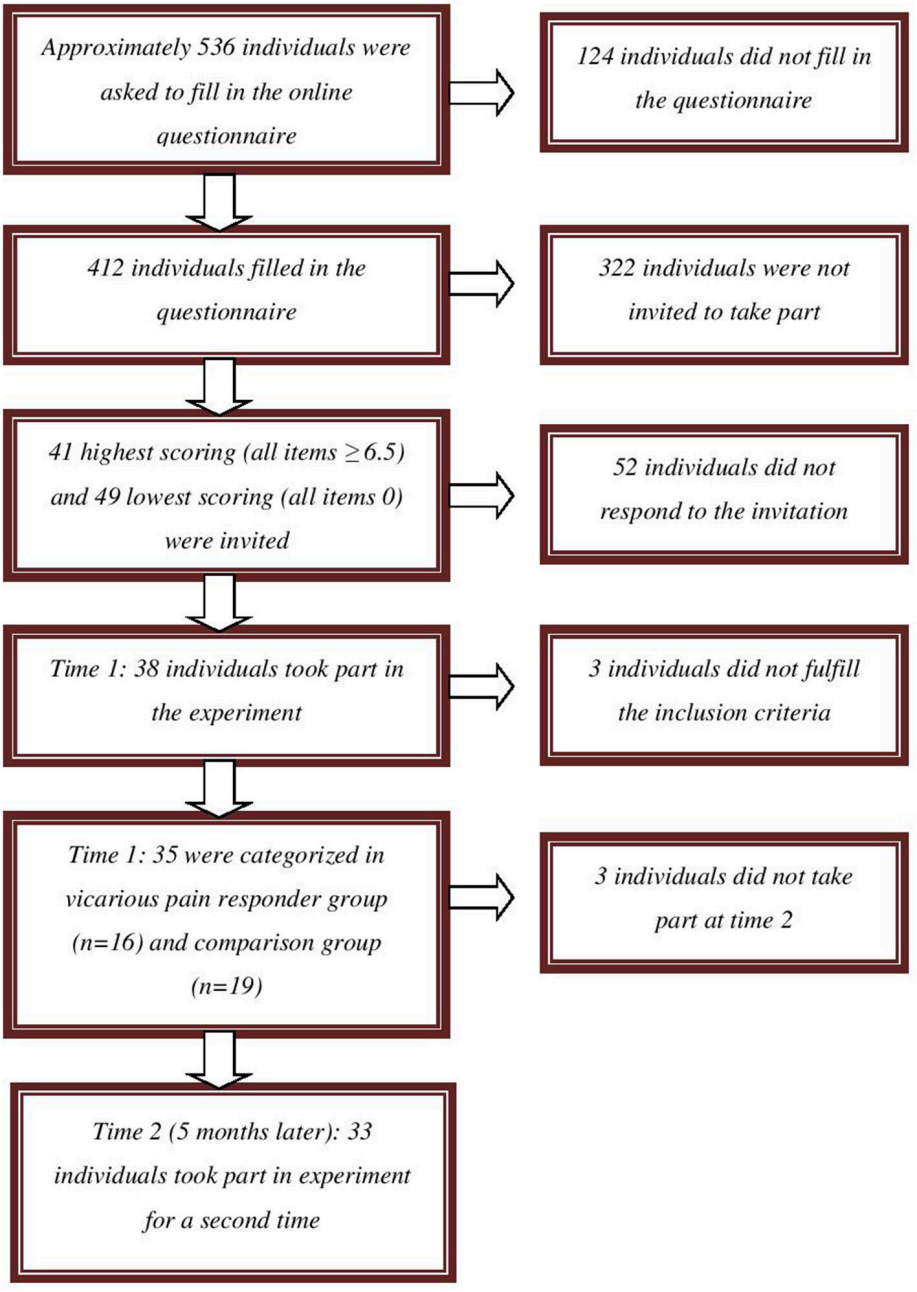
**Flow chart of recruitment of vicarious pain responders and controls**.

Participants were recruited from a pool of approximately 536 undergraduate students from Ghent University who were invited to complete questionnaires screening for, amongst others, the experience of vicarious pain in daily life (October 2012 to February 2013) (see Figure 1). One of these questionnaires assesses the experience of vicarious pain experiences in daily life by means of four items adapted from Banissy et al. (2009). Participants were asked to indicate on an eleven point scale (0–10; totally disagree—totally agree) the extent to which they agreed with the questions: “Do you feel pain in your own body when you see someone accidently bump against the corner of a table?” “Do you have the feeling experiencing pain when you observe another person in pain?” “Do you feel bodily pain when you observe another person in pain?” and “Do you feel a physical sensation (e.g., tingling, stabbing, …) when you observe another person in pain?” Completed questionnaires were available from 412 students. As no standard cut-off for the presence of vicarious pain is available, we invited the highest scoring vicarious pain responders (10%, *n* = 41) over all questions (average score on all items for each individual was ≥ 6.5). This cut-off preserves a balance between extreme values (inviting the highest scoring vicarious pain responders) and a minimum of vicarious pain responders to participate (see Vandenbroucke et al., 2013). We also invited randomly 49 of those who scored 0 on all questions.

In total, 38 undergraduates (34 women) agreed to participate. Their mean age was 19.97 years (*SD* = 3.47, range: 18–36 years). All participants were Caucasian. Participants received either course credits for participation in this experiment (*n* = 21) or were paid (*n* = 17) 10 euro. Participants were categorized in a vicarious pain group and a comparison group based upon the sum of their responses on the items measuring vicarious pain in daily life, administered during the experiment. We considered maintaining all participants whose sum score was ≤15 (*n* = 19; comparison group) and those whose sum score was ≥25 (*n* = 16; vicarious pain responder group) as this cut-off preserves a balance between extreme values (the most extreme scoring vicarious pain responders) and a minimum of vicarious pain responders to analyze. Three participants scoring between 15 and 25 were excluded from the analyses (see Vandenbroucke et al., 2013). Mean age was 20 years in the vicarious pain responder group (*SD* = 4.35, range: 18–36 years) and 20.21 years (*SD* = 2.90, range: 18–29 years) in the comparison group. Of all included participants, one indicated to have experienced an episode of chronic pain during the past 6 months (pain duration was 90 days). This participant was not excluded for participation. Ethical approval was obtained from the Ethics committee of the Faculty of Psychology and Educational Sciences of Ghent University (Belgium).

Approximately 5 months later (Time 2), participants were invited by phone for their participation in a second part, which was described as a subsequent phase of the first experiment in which they participated. The categorization of participants based upon their vicarious pain report in daily life at Time 1 was maintained^1^. The two non-participating individuals were two vicarious pain responders. Thirty-three of the 35 participants (94%; 29 women) agreed to participate a second time. These participants did not make many vicarious errors at time 1 (*n* = 0 and *n* = 3 respectively). Mean age of the participating group was 20.68 years (*SD* = 3.85, range: 18–37 years). All participants were paid 20 euro for their second participation.

### Apparatus and stimuli

#### Somatosensory stimuli

Vibrotactile stimuli (50 Hz, 50 ms) were delivered by means of two resonant-type tactors (C-2 tactor, Engineering Acoustics, Inc.) consisting of a housing that was 3.05 cm in diameter and 0.79 cm high, with a skin contactor that was 0.76 cm in diameter. The somatosensory stimuli were delivered on the skin between thumb and index finger. Through a self-developed software program that was used to control the tactors, all stimulus characteristics (amplitude, duration, and frequency) were entered. The threshold intensity level was individually determined prior to the experiment for each participant (see Procedure-Preparation phase). Both hands were placed on the table in front of the screen and covered of sight by means of a carton box. Four different series of 20 stimuli/trials (two series for each hand) were randomly administered (80 stimuli/trials in total). First, a visual stimulus (an “X” in the middle of the screen, 1000 ms duration) was presented combined with a somatosensory stimulus on the left or right hand. Participants were instructed to report whether they felt a somatosensory stimulus (“yes” or “no”), which was coded by the experimenter by pressing the corresponding response button. Each series started with a stimulus of 0.068 W. The intensity was decreased by 0.0002 W whenever the participants reported feeling a stimulus, and increased by 0.0002 W when no sensation was reported. This resulted, after 80 trials, in a threshold intensity for each hand, which was based upon the mean intensity of the last stimuli of the two series for that particular hand. From this threshold intensity, 1/8 was subtracted (subthreshold) and added to the threshold (above threshold) which resulted in four different intensities (sub and above threshold, one for each hand; see Press et al., 2004). Thresholds for left and right hand were not significantly different at T1 [*t*_(34)_ = 0.69, *p* = 0.50], (threshold left hand: *M* = 0.038 W, *SD* = 0.004 W, range = 0.008 W–0.133 W; threshold right hand: *M* = 0.033 W, *SD* = 0.004 W, range = 0.008 W–0.124 W) and at T2 [*t*_(32)_ = 0.87, *p* = 0.39], (threshold left hand: *M* = 0.033 W, *SD* = 0.004 W, range = 0.006 W–0.133 W; threshold right hand: *M* = 0.029 W, *SD* = 0.004 W, range = 0.003 W–0.089 W).

#### Visual stimuli

**Figure 2:**
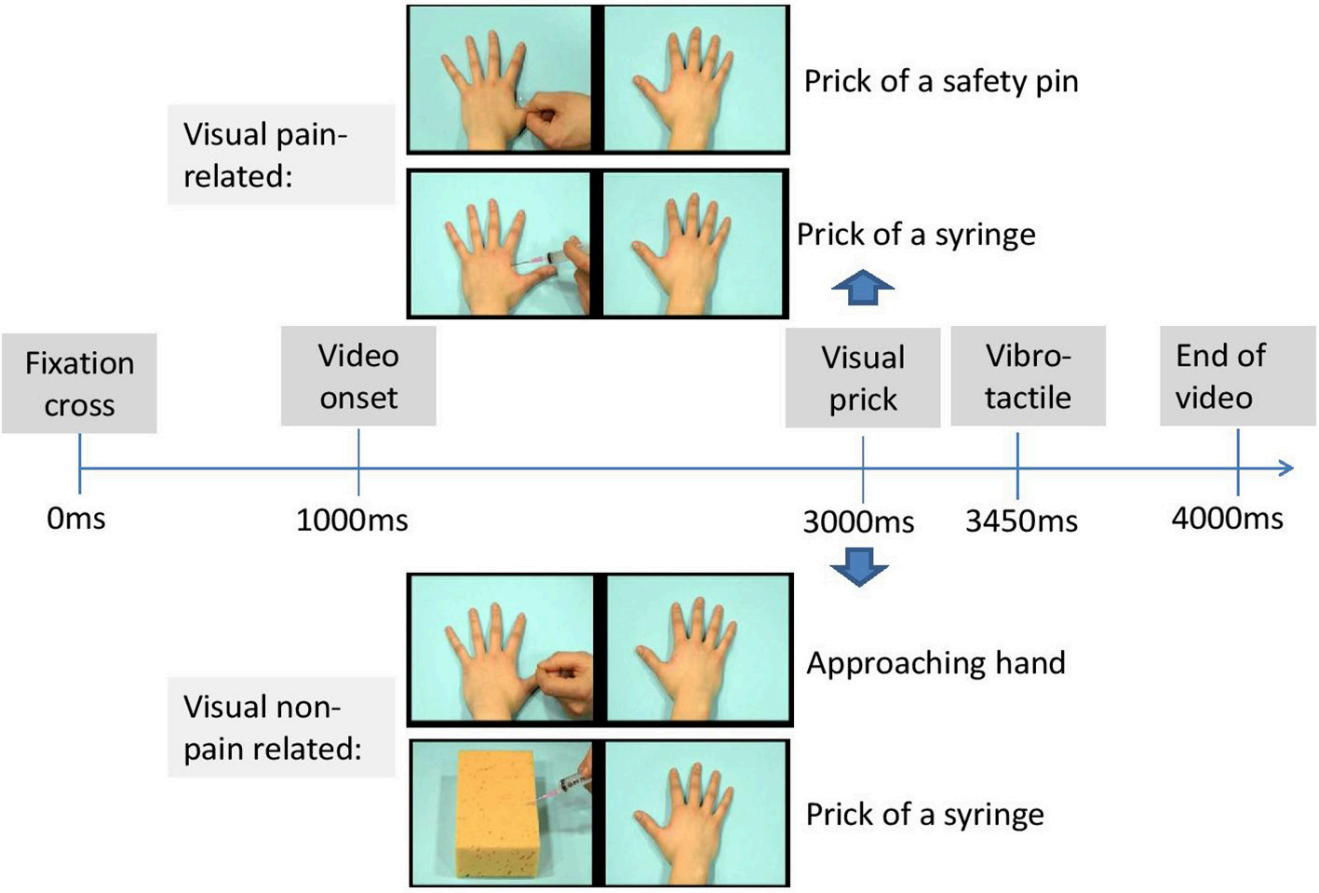
**Timeline of a possible trial**.

The visual stimuli consisted of videos from two categories (pain-related vs. non-pain related), each with a duration of 3 s. The pain-related category included two scenes depicting a left and right hand. One of the two hands was pricked by a syringe (scene 1) or safety pin (scene 2) 2000 ms after video onset. The non-pain related category also consisted of 2 scenes. In the first scene, a left and right hand was presented in which one of these hands was approached by a hand that was not holding an object. In the first scene, a left and right hand was presented in which one of these hands was approached by a hand that was not holding an object. That way, we wanted to control for the motor movement (the same action is performed as in the first category of videos). In the second scene, one hand (left or right) was still present as in all other scenes mentioned above, but at the other site no second hand but a sponge was being pricked by a syringe (see Figure 2). That way, we wanted to control for the possible aversion for the presence of the syringe. The penetration took place also after 2000 ms. The different scenes and the location of the sponge and movement were counterbalanced across videos. The location of the penetration (left vs. right hand) and type of category were counterbalanced across videos. Videos were presented by INQUISIT Millisecond software (http://www.millisecond.com) on a Dell computer with a 19-inch CRT-monitor.

### Self report measures

The Dutch version of the Pain Vigilance and Awareness Questionnaire (PVAQ; Mc Cracken, 1997; Roelofs et al., 2002) was used to measure vigilance to pain. This questionnaire consists of 16 items assessing awareness, consciousness and vigilance to pain on a six-point scale (0 = never; 5 = always). Higher scores on the PVAQ are indicative of greater pain-related vigilance and awareness. The questionnaire can be used in both clinical (Mc Cracken, 1997; Roelofs et al., 2003) and non-clinical (McWilliams and Asmundson, 2001; Roelofs et al., 2002) samples. The Dutch version of the PVAQ is reliable and valid (Roelofs et al., 2002, 2003). Cronbach's alpha for the present study was 0.88 at Time one and 0.95 at Time two.

The Dutch version of the Interpersonal Reactivity Index assessed empathic disposition (IRI; Davis, 1983; De Corte et al., 2007). This questionnaire includes 28 items and consists of 4 subscales: perspective Taking (i.e., cognitively taking the perspective of another, e.g., “I sometimes try to understand my friends better by imagining how things look from their perspective.”), Fantasy (i.e., emotional identification with characters in books, movies etc., e.g., “When I watch a good movie, I can very easily put myself in the place of a leading character.”), Empathic Concern (i.e., feeling emotional concern for others, e.g., “I am often quite touched by things that I see happen.”) and Personal Distress (i.e., negative feelings in response to the distress of others, e.g., “When I see someone who badly needs help in an emergency, I go to pieces.”). Each item is answered on a scale ranging from 1 (“does not describe me very well”) to 5 (“describes me very well”). This questionnaire has shown to be reliable and valid (Davis, 1983; De Corte et al., 2007). Cronbach's alpha's in the current study were 0.77 (empathic concern), 0.77 (personal distress), and 0.55 (perspective taking) and 0.40 (fantasy scale) for Time one. Perspective taking and Fantasy scale were omitted from the analyses because of the low reliability score. At time two, Cronbach's alpha's were 0.81 (empathic concern), 0.77 (personal distress), 0.85 (fantasy scale) and 0.66 (perspective taking). Only those scales showing sufficient reliability at both time moments were maintained in analyses, i.e., hypervigilance for pain, empathic concern and personal distress. Vicarious pain experiences during daily life were measured by means of four items adapted from Banissy et al. (2009) as described in the participants section. In the present study Cronbach's alpha was 0.97 at Time 1.

### Procedure

After signing the informed consent, the participants were seated in front of a table, at about 60 cm away from the computer screen.

#### Preparation phase

First, the detection threshold was determined for each hand separately. The participants were informed that during the experiment they would feel subtle stimuli varying in intensity and length, on their left, right, or both hands and that different videos would be presented which they needed to watch attentively. A carton box covered the hands of the participants which were placed upon the table. The participants were told that the intensity of the somatosensory stimuli could vary across hands and that also trials without any stimulus would be included. In reality, only two fixed predetermined intensities with a fixed duration were applied (threshold intensity ± 1/8) for each hand.

#### Experiment phase

Each trial began with a fixation cross (1000 ms duration) presented in the middle of the computer screen. Next, one of the scenes was presented. In 75% of the trials, a tactile stimulus was delivered 2450 ms after video onset either on the left hand, the right hand, or on both hands of the participant. The somatosensory stimulus was administered with a delay (450 ms after the visual stimulus of penetration of the needle), in line with Banissy and Ward (2007). As such, the following trial types were created: congruent trials, incongruent trials, trials without tactile stimuli, and trials with both hands stimulated. In congruent trials, visual and tactile stimuli were presented at the same spatial location (e.g., on the right). In the incongruent trials, the somatosensory and visual stimuli were presented at opposite locations (e.g., one on the left and the other on the right). The experiment started with 8 practice trials.

**Figure 3:**
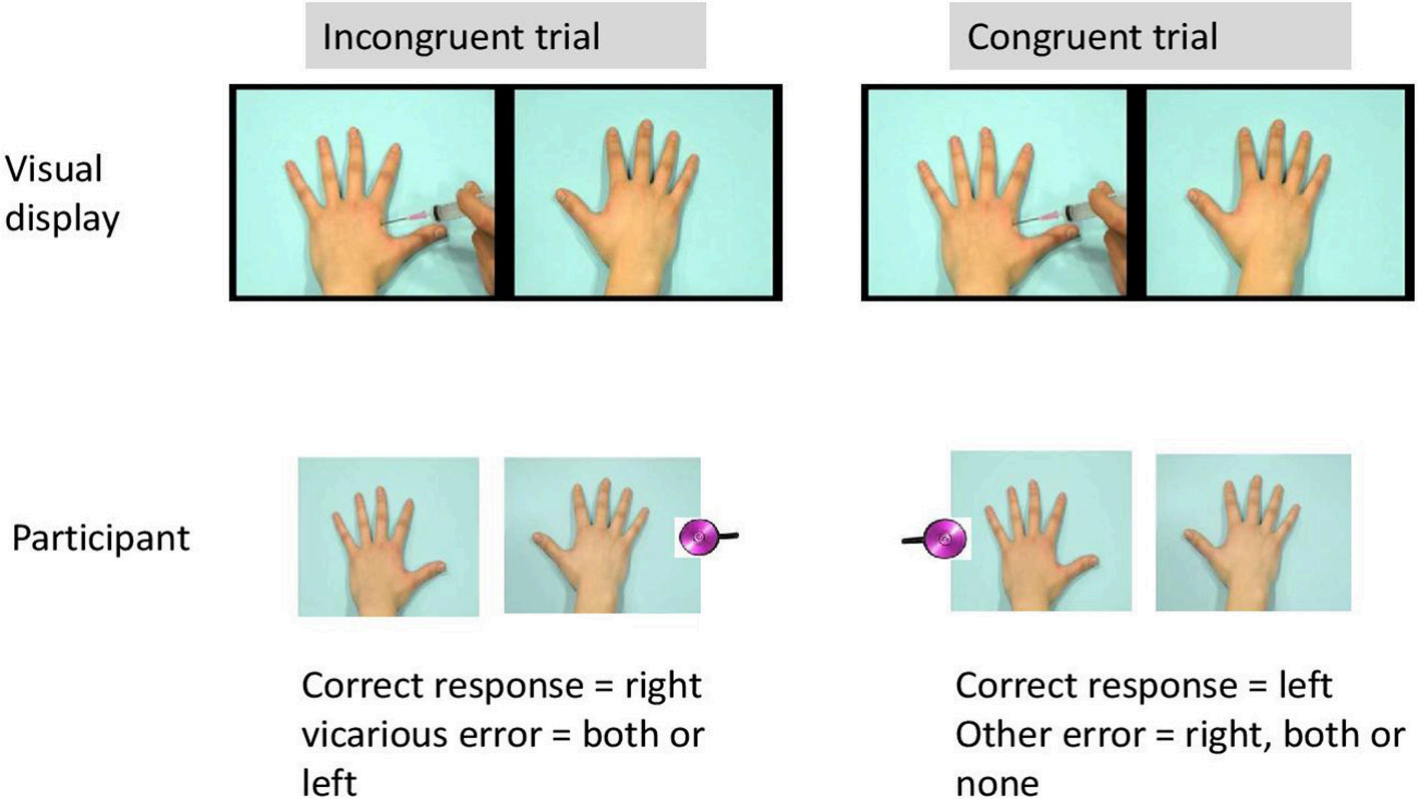
**Example of a possible trial**.

The actual experimental phase consisted of 192 trials divided over three blocks of 64 trials. There were 48 congruent trials, 48 incongruent trials, 48 trials without sensory stimuli and 48 trials with somatosensory stimuli at both hands. Order of trial types was randomized within each block and the somatosensory stimuli were equally distributed within and over each block with an intensity under and above threshold. An overview of all trial types is presented in Table 1. During each trial, participants were requested to report whether a physical sensation was felt by reporting as rapidly as possible “YES” and to discriminate the spatial location of the somatosensory stimuli by reporting “left,” “right,” or “both” (see Figure 3). After the video had ended and 2000 ms elapsed, the word “next” was presented on the screen (see Figure 2). Then, the experimenter coded the response by pressing the corresponding response button (left, right, both, or no response). In this way, the time to respond was equal for every participant. The experiment took approximately 20 min.

**Table 1:** **Detection accuracy for both groups and all video types**.

**Pooled effects**	**Incongruent trials**				**Congruent trials**				**Trials without tactile stimuli**				**Trials with both hands of participant stimulated**			
**Site reported by participant**	**Correct site**	**Opposite site (= site of visual)**	**Both hands**	**No hands**	**Correct site**	**Opposite site to visual and tactile**	**Both hands**	**No hands**	**Site congruent to visual**	**Opposite site to visual**	**Both hands**	**Correct no hands**	**Visual site**	**Opposite site to visual**	**Correct both hands**	**No hands**
	**Detection accuracy**		**Errors**		**Detection accuracy**		**Errors**		**Detection accuracy**		**Errors**		**Detection accuracy**		**Errors**	
**Visual pain** Vicarous pain responder group	49.31%	10.97% vicarious errors	10.42% vicarious errors	29.32%	77.92%	0.97%	2.36%	18.75%	15.69% vicarious errors	1.81%	0.97%	81.53%	26.81% neglect error	11.25%	48.61%	13.33%
**Visual control** Vicarious pain responder group	46.39%	1.67%	2.92%	49.03%	50.00%	0.42%	2.36%	47.22%	2.22%	1.11%	0.56%	96.11%	17.92% neglect error	13.33%	36.81%	31.94%
**Visual pain** Control group	61.51%	2.19% vicarious errors	3.07% vicarious errors	33.22%	67.43%	0.66%	2.19%	29.71%	4.17% vicarious errors	1.53%	0.55%	93.75%	19.96% neglect error	13.16%	49.45%	17.43%
**Visual control** Control group	48.68%	0.66%	1.21%	49.45%	49.45%	0.33%	1.21%	49.01%	0.44%	1.86%	0.11%	97.59%	15.57% neglect error	14.04%	37.94%	32.46%

#### Post-experiment phase

After the experiment, participants were requested to fill out self-report scales measuring hypervigilance for pain (PVAQ), vicarious pain and empathic disposition (IRI). The same procedure was performed at time 2.

### Statistical analysis

#### Vicarious errors

False alarms were calculated from the incongruent trials and from the trials without any somatosensory stimuli when erroneously a somatosensory stimulus was reported in the same spatial location as the visual stimulus. These false alarms were labeled “vicarious experiences” or “vicarious errors” when the visual stimulus contained pain-related information. First, we tested whether the number of false alarms was dependent upon the category of video. As all participants observed both categories of videos and the number of false alarms during both categories of movies were not normally distributed, non-parametric analyses for related samples (Wilcoxon) were used. The number of vicarious experiences was further selected as the dependent variable, as we were particularly interested in those false alarms during pain-related videos (= vicarious errors). To test whether group predicted the number of vicarious errors, count regression models were applied as the use of linear models is considered less appropriate (Vives et al., 2006) when the frequency of responses has a skewed distribution that violates the normality assumption. The basic model to analyze count data is poisson regression, but the variance of counts is often larger than the mean (overdispersion). The Negative Binomial (NB) regression, a Poisson regression with an overdispersion, may therefore better fit the data (e.g., Gardner et al., 1995). As count data may additionally exhibit a lot of zero counts, zero-inflated extensions of both models, called Zero-Inflated Poisson (ZIP) and Zero-Inflated NB (ZINB) models have been developed (see Karazsia and van Dulmen, 2010; Loeys et al., 2012). Deviance tests and the Vuong test were used to select the best fitting count distribution for the dependent variable. A model with “group” as predictor was added, after the best fitting count model was chosen. In a further exploration of the data, hypervigilance for pain, and dispositional empathy and their interaction with group were added in separate models to test whether they had a moderating role. Dummy coding was used for the categorical variables. Regression coefficients are exponentiated (eB) and called Rate Ratios (RRs). In percentages—100 × (eB−1)—RRs reflect the percentage decrease (RR < 1) or increase (RR > 1) in the expected frequency of vicarious errors for each 1-unit increase in the continuous predictor. Same statistical analyses were performed at Time 2. To measure the stability of the vicarious errors, generalized linear mixed models assuming the same count distribution as at the single time moments were applied. As both time moments were included for the same participants, a random intercept was used to capture the dependency within participants. R (version 2.15.1) was used to fit the count models.

#### Detection accuracy

The proportion of correct responses (left vs. right) for congruent and incongruent trials for each category of visual information was calculated (pain-related vs. non-pain related), to investigate whether the observation of pain-related and non-pain related scenes modulated the detection of tactile stimuli. Detection accuracy was measured by means of a 2 (video category: pain-related vs. non-pain related) × 2 (congruency: congruent vs. incongruent) repeated measures ANOVA, with congruency and type of video entered as within-participant variables and “group” as between-subject variable. In a further exploration of the data, hypervigilance for pain and dispositional empathy were added as a covariate in separate models to test whether they had a moderating role. Same statistical analyses were performed at Time 2. Subsequently, to analyze the stability of detection accuracy, Repeated Measure ANOVAs were again executed with the inclusion of an extra within-variable Time (Time 1 vs. Time 2). Repeated measure ANOVAs were conducted with an alpha < 0.05, using SPSS statistical software, version 21.0 for Windows.

#### Neglect errors

The number of neglect errors was calculated based upon those trials in which both hands were stimulated, defined as reporting only the site congruent to the visual information and missing the actual tactile stimuli on both hands. To test whether the number of neglect errors was dependent upon the category of video, non-parametric analyses for related samples (Wilcoxon) were used. Count regression models were applied in which the dependent variable was the number of neglect errors during pain-related visual information. After the best fitting count model was chosen, a model with “group” as predictor was added. In a further exploration of the data, hypervigilance for pain and dispositional empathy and their interaction with group were added in separate models to test whether they had a moderating role. Same statistical analyses were performed at Time 2. To measure the stability of the neglect errors, generalized linear mixed models for count data were applied as described above in the section of the vicarious errors.

## Results

### Descriptives

Mean scores, standard deviations and correlations at Time 1, 2 and both time moments are presented in Tables 2, 3. For variables that were not normally distributed (Kolmogorov-Smirnoff, *p* < 0.05), Spearman correlations were computed.

**Table 2:** **Pearson/Spearman correlations of all measures (T1, T2 and pooled effects)**.

	**Time 1**					**Time 2**				**Pooled effects**			
	**2**	**3**	**4**	**5**	**6**	**2**	**3**	**4**	**5**	**2**	**3**	**4**	**5**
1. Vicarious errors	0.45^**^	0.32	0.37^*^	0.38^*^	0.39^*^	0.36^*^	0.22	0.20	0.08	0.41^**^	0.26^*^	0.29^*^	0.22
2. Neglect errors (pain-related videos)	−	0.14	0.20	0.01	0.20	−	0.23	0.18	0.17	−	0.17	0.11	0.08
3. Hypervigilance (PVAQ)		−	0.32	0.16	0.52^**^		−	0.11	0.18		−	0.22	0.17
4. Empathic concern			−	−0.09	0.33			−	0.17			−	0.06
5. Personal distress				−	0.39^*^				−				−
6. Vicarious pain during daily life					−								

Pain Vigilance and Awareness Questionnaire (PVAQ).

* p < 0.05;

** p < 0.01.

**Table 3:** **Mean scores and standard deviations of all measures (T1, T2 and pooled effects)**.

	**Time 1**			**Time 2**			**Pooled effects**		
	***M* (*SD*) vicarious pain responders**	***M* (*SD*) controls**	***M* (*SD*) total group**	***M* (*SD*) vicarious pain responders**	***M* (*SD*) controls**	***M* (*SD*) total group**	***M* (*SD*) vicarious pain responders**	***M* (*SD*) controls**	***M* (*SD*) total group**
1. Vicarious errors	6.56 (10.94)	1.84 (2.34)	4.00 (7.84)	11.57 (15.23)	2.68 (3.32)	6.45 (10.97)	8.90 (13.13)	2.26 (2.86)	5.19 (9.50)
2. Neglect errors (pain-related videos)	6.31 (3.93)	5.05 (2.70)	5.63 (3.33)	6.57 (4.07)	4.53 (2.25)	5.39 (3.26)	6.43 (3.93)	4.79 (2.46)	5.51 (3.27)
3. Hypervigilance (PVAQ)	44.25 (10.59)	33.95 (11.16)	38.66 (11.94)	46.94 (11.58)	34.53 (14.88)	39.79 (14.76)	45.51 (10.95)	34.24 (12.98)	39.21 (13.29)
4. Empathic concern	20.63 (3.32)	17.21 (4.91)	18.77 (4.54)	21.36 (3.73)	17.79 (4.91)	19.30 (4.73)	20.97 (3.48)	17.50 (4.85)	19.03 (4.61)
5. Personal distress	14.75 (4.54)	13.05 (3.72)	13.83 (4.14)	15.36 (4.53)	13.66 (4.00)	14.38 (4.25)	15.03 (4.47)	13.36 (3.82)	14.10 (4.17)
6. Vicarious pain during daily life	28.88 (4.00)	6.37 (5.10)	16.66 (12.25)						

Pain Vigilance and Awareness Questionnaire (PVAQ).

At both time points, a significant difference was found between both groups in empathic concern [time 1: *t*_(33)_ = −2.36, *p* = 0.02; time 2: *t*_(31)_ = −2.28, *p* = 0.03] and PVAQ [time 1: *t*_(33)_ = −2.79, *p* < 0.01; time 2: *t*_(31)_ = −2.59, *p* = 0.01]. The vicarious pain responder group was more empathic concerned and more hypervigilant for pain.

### Vicarious errors

#### Time 1. The effect of group on vicarious errors moderated by hypervigilance and empathic concern

A main effect of video category upon the presence of false alarms was found (Wilcoxon, *p* < 0.01), indicating that participants more often reported false alarms when the visual stimulus contained pain-related information. In 8.33% of the trials vicarious errors were made (140 vicarious errors from a total of 1680 trials), mainly in the vicarious pain responder group (75% of all vicarious errors; *n* = 105). Two participants in the vicarious pain responder group were responsible for 62.86% of all vicarious errors (66 of a total of 105 vicarious errors). Results based on negative binomial regression models further showed a main effect of group; i.e., the number of vicarious errors was 256% higher in the vicarious pain responder group compared with the comparison group (*RR* = 3.56; *p* = 0.005). No interactions were found between group and Personal distress (*p* = 0.12). A significant interaction was found between group and PVAQ (*p* = 0.02). For vicarious pain responders, the number of vicarious errors decreased by 5% (*RR* = 0.95) for every 1-unit increase in hypervigilance for pain. For the comparison group, the number of vicarious errors increased by 5% (*RR* = 1.05) for every 1-unit increase in hypervigilance for pain. Also a significant interaction was found between group and empathic concern (*p* = 0.003). For the comparison group, the number of vicarious errors decreased by 2% (*RR* = 0.98) for every 1-unit increase in empathic concern. For vicarious pain responders, the number of vicarious errors increased by 36% (*RR* = 1.36) for every 1-unit increase in empathic concern measured at Time 1 (see Table 4).

**Table 4:** **Rate Ratio and Confidence Intervals for neglect errors and vicarious errors (T1, T2 and pooled effects)**.

**Variables**		**Vicarious errors**			**Neglect errors**		
		**Time 1 RR (95% CI)**	**Time 2 RR (95% CI)**	**Pooled effects RR (95% CI)**	**Time 1 RR (95% CI)**	**Time 2 RR (95% CI)**	**Pooled effects RR (95% CI)**
Group^a^		3.56^**^ [1.47, 8.65]	4.31^**^ [1.76, 10.55]	2.77^*^ [1.14, 6.69]	1.25 [0.86, 1.81]	1.45 [0.98, 2.16]	1.32 [0.95, 1.82]
Time^b^				1.61^*^ [1.03, 2.52]			0.97 [0.79, 1.19]
Hypervigilance	VPR	0.95 [0.90, 1.01]	1.03 [0.97, 1.09]	1.01 [0.97, 1.05]	0.99 [0.95, 1.02]	1.01 [0.99, 1.04]	1.00 [0.99, 1.02]
	C	1.05 [0.99, 1.10]	1.00 [0.96, 1.04]	1.01 [0.98, 1.04]	1.00 [0.98, 1.03]	1.00 [0.98, 1.02]	1.00 [0.98, 1.01]
	VPR vs. C	0.91^*^ [0.84, 0.98]	1.03 [0.96, 1.10]	1.00 [0.95, 1.05]	0.99 [0.95, 1.02]	1.01 [0.98, 1.04]	1.00 [0.98, 1.03]
Empathic concern	VPR	1.36^***^ [1.13, 1.62]	1.11 [0.93, 1.32]	1.16 [1.00, 1.35]	1.08^*^ [1.01, 1.16]	1.02 [0.94, 1.10]	1.04 [0.98, 1.11]
	C	0.98 [0.87, 1.10]	1.03 [0.91, 1.17]	0.98 [0.88, 1.10]	0.96 [0.92, 1.00]	1.00 [0.95, 1.06]	0.98 [0.94, 1.02]
	VPR vs. C	1.39^**^ [1.12, 1.72]	1.07 [0.86, 1.33]	1.18 [0.98, 1.42]	1.13^**^ [1.04, 1.23]	1.02 [0.92, 1.12]	1.06 [0.99, 1.14]
Personal distress	VPR	1.10 [0.97, 1.25]	0.97 [0.83, 1.12]	1.01 [0.89, 1.14]	1.00 [0.95, 1.06]	0.99 [0.93, 1.05]	0.98 [0.93, 1.03]
	C	1.35^**^ [1.08, 1.70]	1.04 [0.89, 1.21]	1.08 [0.93, 1.25]	1.06 [0.98, 1.14]	1.07 [1.00, 1.15]	1.05 [0.99, 1.10]
	VPR vs. C	0.81 [0.62, 1.05]	0.93 [0.75, 1.16]	0.93 [0.77, 1.13]	0.95 [0.87, 1.04]	0.92 [0.84, 1.01]	0.94 [0.87, 1.00]

a Reference category is the comparison group.

b Reference category is time 1.

RR, rate ratios (e^B^); CI, confidence interval; VPR, vicarious pain responders; C, controls.

* p < 0.05;

** p < 0.01;

*** p < 0.001.

#### Time 2. The effect of group on vicarious errors, but no moderation

Again, a main effect of video category upon the presence of false alarms was found (Wilcoxon, *p* < 0.01). indicating that participants more often reported false alarms when the visual stimulus contained pain-related information. In 13.45% of the trials vicarious errors were made (213 vicarious errors from a total of 1584 trials), mainly in the vicarious pain responder group (76.06% of all vicarious errors; *n* = 162). Four participants in the vicarious pain responder group were responsible for 82.72% for all vicarious errors (134 of a total of 162 vicarious errors). Negative binomial regression models revealed that the number of vicarious errors was again dependent upon group (*p* = 0.001). The vicarious pain responder group made 331% more vicarious errors than the comparison group (*RR* = 4.31). No interactions were found between group and personal distress (*p* = 0.54), empathic concern (*p* = 0.53) and hypervigilance for pain (*p* = 0.44) measured at Time 2 (see Table 4).

#### Stability of vicarious errors. The effect of group on vicarious errors, but no moderation

In line with Time 1 and Time 2 results, a main effect of video category upon the presence of false alarms was found (Wilcoxon, *p* < 0.01). In 10.81% of the trials vicarious errors were made (353 vicarious errors from a total of 3264 trials), mainly in the vicarious pain responder group (75.64% of all vicarious errors; *n* = 267). Four participants in the vicarious pain responder group were responsible for 59% for all vicarious errors at both moments (209 of a total of 353 vicarious errors). Two of these four vicarious pain responders showed a lot of vicarious errors at time two (35 and 32 vicarious errors respectively) but did not show this pattern at time one (4 and 5 vicarious errors respectively). The other two vicarious pain responders showed a relative stable number of vicarious errors (T1: *n* = 28 and *n* = 38; T2: *n* = 45 and *n* = 22).

The generalized linear mixed model assuming a negative binomial distribution revealed a main effect of time (*p* = 0.04), with vicarious errors increasing with 61% at time two (*RR* = 1.61). Also, the number of vicarious experiences was, across Time 1 and Time 2, dependent upon group (*p* = 0.02). Vicarious pain responders made 177% more vicarious errors compared with controls (*RR* = 2.77). No interaction occurred between group and time (*p* = 0.66). Group did not significantly interact with hypervigilance for pain (*p* = 0.99), empathic concern (*p* = 0.07) or personal distress (*p* = 0.46) measured at both time moments (see Table 4).

### Detection accuracy

#### Time 1. The effect of video and congruency on detection accuracy moderated by group

In line with our hypotheses, a 2 (video category: pain-related vs. non-pain related) × 2 (congruency: congruent vs. incongruent) repeated measures ANOVA with “group” (vicarious pain responder vs. comparison group) as between-subject variable showed a main effect of video category [*F*_(1, 33)_ = 38.31, *p* < 0.0001, Cohen's *d* = 1.02, (95% CI:0.63, 1.42)]. Pain-related videos resulted in a better detection of vibrotactile stimuli compared with non-pain related videos. Also a main effect for congruency was found [*F*_(1, 33)_ = 12.83, *p* = 0.001, Cohen's *d* = 0.49, (95% CI:0.20, 0.78)]. An interaction occurred between congruency and video category: the CCE was dependent upon the type of video presented [*F*_(1, 33)_ = 24.96, *p* < 0.0001, Cohen's *d* = −0.84, (95% CI:-1.27, −0.41)]. A paired sample *t*-test showed the CCE was only significant for the pain-related videos [*t*_(34)_ = −4.36, *p* < 0.001, Cohen's *d* = 0.78, (95% CI:0.37, 1.18)] and not for the non-pain related videos [*t*_(34)_ = 0.12, *p* = 0.91, Cohen's *d* = 0.01, (95% CI: −0.21, 0.24.)]. A significant interaction was found between group, video and congruency [*F*_(1, 33)_ = 6.39, *p* = 0.02], showing that the interaction between video and congruency was only significant for the vicarious pain responder group [*F*_(1, 15)_ = 23.44, *p* < 0.001, Cohen's *d* = −1.63, (95% CI: −2.64, −0.62)]. In the comparison group, detection accuracy during pain-related and non-pain related was independent of congruency. No main effect occurred for group [*F*_(1, 33)_ = 0.21, *p* = 0.65, Cohen's *d* = −0.39, (95% CI: −0.80, 0.02)]. No interaction was found between group and video [*F*_(1, 33)_ = 0.07, *p* = 0.79] and between group and congruency [*F*_(1, 33)_ = 3.45, *p* = 0.07]. No moderating role was found of hypervigilance or dispositional empathy measured at Time 1 (all *p* > 0.05) (see Table 5).

**Table 5:** **The significant effects of video, congruency and group on detection accuracy and Confidence intervals (T1, T2 and pooled effects)**.

**Variables**	**Detection accuracy**		
	**Time 1**	**Time 2**	**Pooled effects**
Video	1.02^***^[0.63, 1.42]	0.08^***^(0.48, 1.11]	1.09^***^[0.72, 1.45]
Congruency	0.49^**^[0.20, 0.78]	0.54^***^[0.18, 0.90]	−0.62^***^[−1.08, −0.17]
Congruency^*^Video	−0.84^***^[−1.27, −0.41]	0.66^**^[0.19, 1.13]	−0.75^***^[−1.11, −0.38]
Congruency^*^Video^*^group VPR	−1.63^***^[−2.64, −0.62]	−1.28^**^[−2.13, −0.44]	−0.98^***^[−1.44, −0.53]
C	−0.39 [−0.80, 0.02]	−0.13 [−0.62, 0.36]	−0.54 [−1.16, 0.08]

VPR, vicarious pain responders; C, controls.

* p < 0.05;

** p < 0.01;

*** p < 0.001.

#### Time 2. The effect of video and congruency on detection accuracy moderated by group

In line with Time one, a main effect of video category was found [*F*_(1, 31)_ = 30.19, *p* < 0.0001, Cohen's *d* = 0.80, (95% CI:0.48, 1.11)]; the observation of pain-related videos resulted in a better detection of vibrotactile stimuli compared with non-pain related videos. Also a main effect for congruency occurred [*F*_(1, 31)_ = 15.86, *p* < 0.001, Cohen's *d* = 0.54, (95% CI:0.18, 0.90)]. An interaction occurred between congruency and video category: the CCE was dependent on the type of video presented [*F*_(1, 31)_ = 14.59, *p* = 0.001, Cohen's *d* = 0.66, (95% CI:0.19, 1.13)]. A paired sample *t*-test showed the CCE was only significant for the pain-related videos [*t*_(32)_ = −3.41, *p* = 0.002, Cohen's *d* = 0.79, (95% CI:0.25, 1.32)]. A significant interaction was found between group, video category and congruency [*F*_(1, 31)_ = 10.30, *p* = 0.003]. The interaction between video category and congruency was only significant for the vicarious pain responder group [*F*_(1, 13)_ = 16.17, *p* = 0.001, Cohen's *d* = −1.28, (95% CI: −2.13, −0.44)]. No main effect occurred for group [*F*_(1, 31)_ = 0.56, *p* = 0.46]. An interaction was found between group and congruency [*F*_(1, 31)_ = 9.10, *p* = 0.005], indicating that the congruency effect was only present in the vicarious pain responder group [*F*_(1, 13)_ = 10.59, *p* = 0.006]. No interaction was found between group and video category [*F*_(1, 31)_ = 0.11, *p* = 0.75]. No moderating role was found of the individual difference variables measured at Time 2 (all *p* > 0.05) (see Table 5).

#### Stability of detection accuracy. Effect of video and congruency on detection accuracy moderated by group

**Figure 4:**
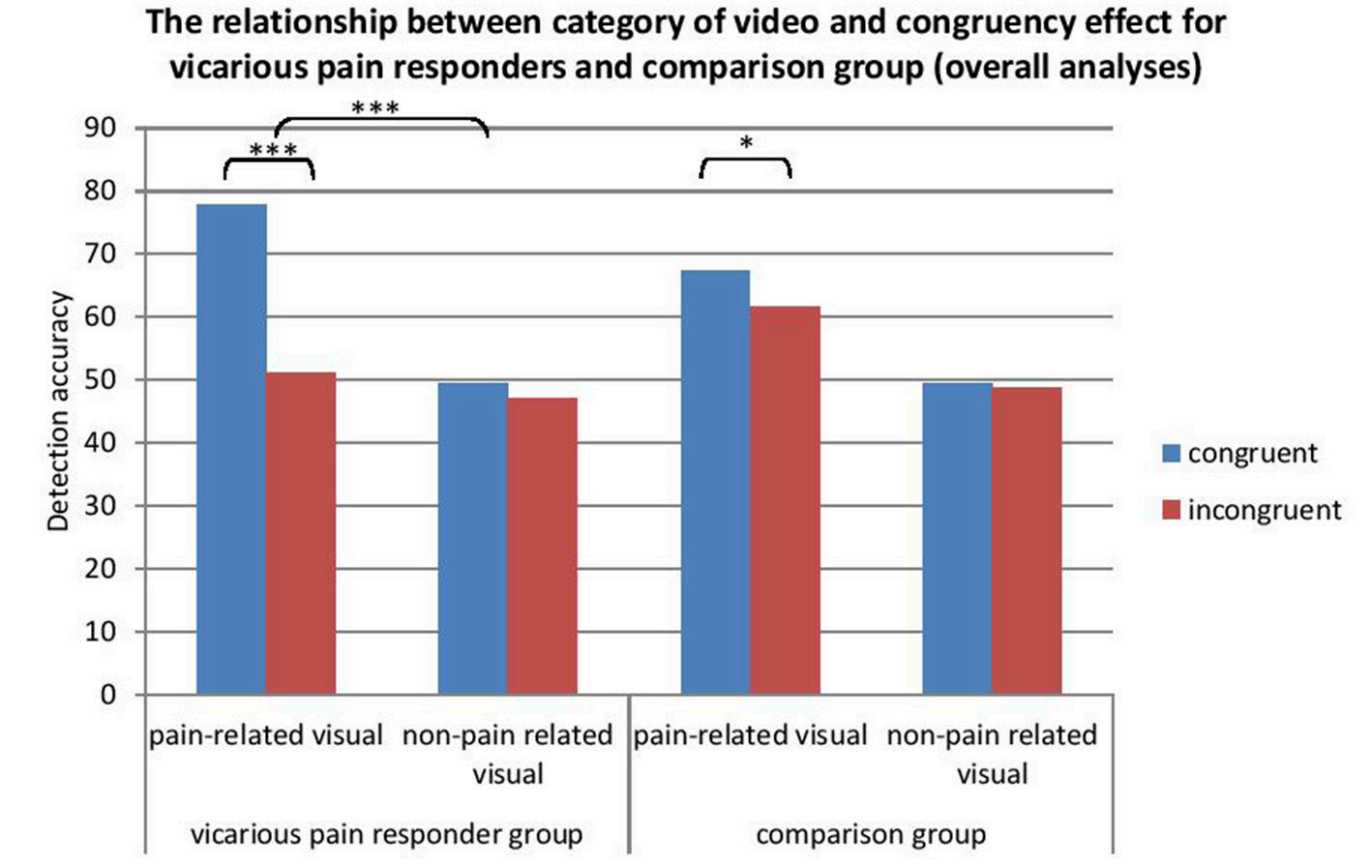
**The relationship between video category and congruency for vicarious pain responders and controls (pooled effects)**. ^*^p < 0.05; ^***^p < 0.001.

Overall results across time showed a main effect for video category [*F*_(1, 31)_ = 46.35, *p* < 0.001, Cohen's *d* = 1.09, (95% CI:0.72, 1.45)] in which pain-related videos resulted in a better detection compared with non-pain related videos. Also a main effect of congruency occurred [*F*_(1, 31)_ = 25.81, *p* < 0.0001, Cohen's *d* = −0.62, (95% CI: −1.08, −0.17)]. An interaction occurred between congruency and video category: the CCE was dependent on the type of video presented [*F*_(1, 31)_ = 30.40, *p* < 0.0001, Cohen's *d* = −0.75, [95% CI: −1.11, −0.38]). A paired sample *t*-test showed the CCE was only significant for the pain-related videos [*t*_(67)_ = −5.41, *p* < 0.001, cohen's *d* = −0.80, (95% CI: −1.14, −0.47)] and not for the non-pain related videos [*t*_(67)_ = −1.24, *p* = 0.22, cohen's *d* = −0.11, (95% CI: −0.27, 0.06)]. No main effect occurred for group [*F*_(1, 31)_ = 0.97, *p* = 0.77] and time [*F*_(1, 31)_ = 0.09, *p* = 0.77]. An interaction was found between group and congruency [*F*_(1, 31)_ = 12.06, *p* = 0.002]. The congruency effect was present in the vicarious pain responder group [*F*_(1, 13)_ = 17.09, *p* = 0.001] but not in the comparison group [*F*_(1, 18)_ = 4.01, *p* = 0.06]. A significant interaction was found between group, video category and congruency [*F*_(1, 31)_ = 13.50, *p* = 0.001] (Figure 4). The interaction between video category and congruency was only significant for the vicarious pain responder group [*F*_(1, 13)_ = 23.37, *p* < 0.001, cohen's *d* = −0.98, (95% CI: −1.44, −0.53)]. No moderating role was found of any of the individual difference variables measured at both time moments (all > 0.05) (see Table 5).

### Neglect errors

#### Time 1. No effect of group on neglect errors

A main effect of video category upon the presence of neglect errors was found (Wilcoxon, *p* < 0.01), indicating that participants more often tended to neglect the side that was incongruent with the visual stimulus when this latter contained pain-related information. In 23.45% of the trials in which both hands were stimulated during the observation of pain-related stimuli, neglect errors were made (197 from a total of 840 trials). Of all neglect errors, 51.27% (*n* = 101) occurred in the vicarious pain responder group. Results based on negative binomial regression models showed that the number of neglect errors during pain-related visual stimuli was not dependent upon group (*p* = 0.24). No significant interaction was found of group with hypervigilance for pain (*p* = 0.50) and personal distress (*p* = 0.29). A significant interaction was found between empathic concern and group (*p* = 0.004). For the comparison group, the number of neglect errors during pain-related visual stimuli decreased with 4% (*RR* = 0.96) for every 1-unit increase in empathic concern. For the vicarious pain responder group, the number of neglect errors increased with 8% (*RR* = 1.08) for every 1-unit increase in empathic concern measured at Time 1 (see Table 4).

#### Time 2. No effect of group on neglect errors

Again, a main effect of video category upon the presence of neglect errors was found (Wilcoxon, *p* < 0.01) indicating that participants more often tended to neglect the side that was incongruent with the visual stimulus when this latter contained pain-related information. In 22.47% of the trials in which both hands were stimulated during the observation of pain-related stimuli, neglect errors were made (178 from a total of 792 trials).Of all neglect errors 51.69% occurred in the vicarious pain responder group (*n* = 92). Results based on negative binomial regression models showed that the number of neglect errors during pain-related visual stimuli was not dependent upon group (*p* = 0.07). No significant interaction was found of group with hypervigilance (*p* = 0.47), empathic concern (*p* = 0.73) or personal distress (*p* = 0.07) measured at Time 2 (see Table 4).

#### Stability of neglect errors. No effect of group on neglect errors

In line with Time 1 and Time 2 results, a main effect of video category upon the presence of neglect errors was found (Wilcoxon, *p* < 0.01). Neglect errors were made in 22.98% of the trials in which both hands were stimulated during the observation of pain-related stimuli (375 from a total of 1632 trials). Of all neglect errors 51.47% occurred in the vicarious pain responder group (*n* = 193). In contrast with vicarious errors, no large discrepancies occurred in the number of neglect errors during pain-related videos within subjects over time (maximum discrepancy between T1 and T2 was 8 neglect errors). The generalized linear mixed model analysis including Time showed that the number of neglect errors during pain-related videos was independent from group (*p* = 0.10) or time (*p* = 0.76). Also no interaction occurred between group and time (*p* = 0.46). Group did not significantly interact with hypervigilance for pain (*p* = 0.71), empathic concern (*p* = 0.11) or personal distress (*p* = 0.06) measured at both time moments (see Table 4).

## General discussion

This study investigated whether vicarious pain responders (who report vicarious pain in daily life) and controls (comparison group) differ in the report of vicarious experiences and the detection of somatosensory stimuli while observing another in pain during an experimental paradigm. Furthermore, the stability of vicarious experiences was examined. Additionally, we explored the effects of some potential modulators proposed by Fitzgibbon et al. (2010b, 2012), i.e., dispositional empathy and hypervigilance to pain. Participants were presented a series of videos showing hands being pricked and non-pain related information such as a sponge being pricked whilst receiving occasionally near-threshold vibrotactile stimuli themselves. Participants were required to report whether and where they felt a somatosensory stimulus.

Overall, the occurrence of vicarious experiences was low (8.33% at Time 1, 13.45% at Time 2). Nevertheless, the percentage of vicarious errors was larger than those reported by Vandenbroucke et al. (2013; 0.88% in study 1 and 2.7% in study 2) using a highly similar paradigm. A notable difference was the use of vibrotactile stimuli near threshold in the present study, whereas in the study of Vandenbroucke et al. (2013) electrocutaneous stimuli that elicited painful pricking experiences were used. Probably, the vicarious experiences are subtle, vague sensations that are more easily confused with low intense tactile sensations than with painful sensations. This explanation is in line with the study of Osborn and Derbyshire, 2010), who reported that participants most often described vicarious sensations as “tingling.”

Of interest to this study was whether participants who reported vicarious pain experiences in daily life, also displayed more vicarious experiences in an experimental setup. Our results show that this is indeed the case, and therefore extend the results of a previous study (Vandenbroucke et al., 2013). Our study did not only focus upon vicarious experiences, but also a less extreme position, i.e., the modulation of detection of tactile stimuli during the observation of pain-related and non-pain related situations. This objective was accomplished by investigating detection accuracy of vibrotactile stimuli and neglect errors. The observation of pain-related videos facilitated the detection of vibrotactile stimuli. In the present study, vicarious pain responders were also better in detecting vibrotactile stimuli when pain-related videos and vibrotactile stimuli were presented in the same spatial location than when presented in an opposite location. This is consistent with reaction time research of Banissy and Ward (2007) showing that vicarious responders were faster at identifying a site touched on their face or hands when actual touch was congruent with their vicarious touch compared with incongruent trials. This pattern was not found in the study of Banissy and Ward (2007) when participants observed touch to objects. The results in this experiment are consistent with the idea that observing bodily sensations might influence own somatic experiences (Godinho et al., 2006; Jackson et al., 2006; Costantini et al., 2008; Han et al., 2009). Neuroimaging and EEG studies have shown that the observation of touch leads to an enhanced activation in the somatosensory cortices (Blakemore et al., 2005; Schaefer et al., 2006; Ebisch et al., 2008; Pihko et al., 2010; Martinez-Jauand et al., 2012) which could explain the conscious experience of vicarious sensations as these brain regions are more related to interpreting the localization and intensity of a nociceptive stimulus (Bushnell et al., 1999). In our study, both groups evidenced a similar number of neglect errors. Compared with vicarious errors, neglect errors were more frequently made (Time 1: 23.45%; Time 2: 22.47%) and were as common in both groups. This again suggests that the observation of pain-related information may rather give rise to a modulation of somatosensory experiences rather than the pure induction of illusionary experiences.

Furthermore, the phenomenon seemed to be robust: the phenomenon was also observed when these participants performed the experiment a second time, 5 months later. This is an important finding. Often participants who report vicarious experiences in daily life are invited for further investigations at a later time. It is therefore important to know that the phenomenon is stable across time. The general increase of vicarious errors at time two may be due to other factors such as memory processes as participants may recognize the experiment in which they all already participated. Nevertheless, there are some issues that deserve further scrutiny. First, stability was observed at group level, but there was variation at the individual level. We observed that two of the four vicarious pain responders who were responsible for most vicarious experiences at time two (35 and 32 vicarious errors respectively) did not show this pattern at time one (4 and 5 vicarious errors respectively). A similar variability between individuals, but to a lesser extent was observed for individuals from the comparison group. Further research may examine those individuals demonstrating stability in the report of vicarious experiences on a single case level. Probably these individuals may share features in contrast to those showing a variability in the report of vicarious experiences. Second, some models (Fitzgibbon et al., 2010b, 2012) proposed that individual characteristics such as dispositional empathy and hypervigilance are important moderators. Our results regarding these variables are variable and not consistent across time. It may well be that these individual difference variables may be less important than previously suggested. In order to further the research, we propose that authors are transparent about which individual difference variables are assessed, and systematically report these results, albeit that they are not significant (Simmons et al., 2011). That way a publication bias may be prevented, and a strong database for future secondary or meta-analytic analyses may be developed.

Some limitations of the present study deserve further consideration. First, vibrotactile stimuli were administered instead of painful stimuli. This enhanced the occurrence of vicarious experiences which we consequently not labeled as vicarious pain. Second, only few people reported vicarious pain experiences in daily life, resulting in a small sample size. Also, our sample was unbalanced in terms of gender. The number of vicarious pain responders who took part in the experiment was, however, comparable to other studies including participants reporting vicarious bodily sensations (Banissy and Ward, 2007; Osborn and Derbyshire, 2010; Vandenbroucke et al., 2013). Third, it is unclear to what extent the observed effects are specific to observing pain: we did not include videos in which a hand is being touched. Future research may compare the effects of touch videos and pain videos upon several outcomes to disentangle the pain-specific effects upon somatosensation. Previous studies were also not able to disentangle the effects of observing pain vs. touch as they compared observing touch vs. no touch (i.e., a light), human parts being touched vs. the observation of the human body, observing touch and experiencing touch (e.g., Keysers et al., 2004; Blakemore et al., 2005; Serino et al., 2008; Cardini et al., 2011; Gillmeister, 2014). Finally, we included video clips showing hands being pricked. These videos depict less intense pain compared to the images and movies used in the study of Osborn and Derbyshire (2010). Some may argue that vicarious experiences may be elicited more easily when very intense pain is observed. That said, vicarious pain responders in this study reported more vicarious errors during the observation of a subtle injury (the needle prick) as compared with controls, indicating that vicarious experiences can also be observed with low intense pain stimuli.

### Conflict of interest statement

The authors declare that the research was conducted in the absence of any commercial or financial relationships that could be construed as a potential conflict of interest.

## Footnotes

^1^If categorization of Time 2 would be used, 2 controls and 2 vicarious pain responders would be categorized to the other group.
